# Supplementary material for: ICU-Acquired Pneumonia Is Associated with Poor Health Post-COVID-19 Syndrome
Source: J Clin Med. 2021 Dec 31;11(1):224. doi: 10.3390/jcm11010224 (PMC8746263; doi:10.3390/jcm11010224)
Supplement: Supplementary file 1 [file jcm-11-00224-s001.zip › jcm-1507173-supplementary.pdf]

Supplementary Table S1. Additional therapy provided to patients based on respiratory symptoms and clinical resolution.

Variable	Post-COVID	P-value (vs. no persistent post-COVID-19 symptoms
**Any respiratory therapy**	106(83.5)	0.004
**Continuous Home oxygen**	38	<0.001
**SABA**	24(82.8)	0.3
**LABA**	20(87)	0.2
**LAMA**	20(87)	0.3
**Systemic corticosteroids**	31(72.1)	0.8

Abbreviations: SABA: Short-acting beta-agonists (inhaled). LABA: Long-acting beta-agonists (inhaled). LAMA: Long-acting muscarinic antagonist.

Acknowledgements

CIBERESUCICOVID COLLABORATORS

Berta Adell-Serrano, María Aguilar Cabello, Victoria Alcaraz-Serrano, Cesar Aldecoa, Cynthia Alegre, Sergio Álvarez, Antonjo Álvarez Ruiz, Rut Andrea, José Ángel, Marta Arrieta, J Ignacio Ayestarán, Joan Ramon Badia, Mariona Badía, Orville Báez Pravia, Ana Balan Mariño, Begoña Balsera, Laura Barbena, Enric Barbeta, Tommaso Bardi, Patricia Barral Segade, Marta Barroso, José Ángel Berezo García, Judit Bigas, Rafael Blancas, María Luisa Blasco Cortés, María Boado, María Bodi Saera, Neus Bofill, María Teresa Bouza Vieiro, Leticia Bueno, Juan Bustamante-Munguira, David Campi Hermoso, Sandra Campos Fernández, Iosune Cano, Maria Luisa Cantón-Bulnes, Pablo Cardina Fernández, Laura Carrión García, Sula Carvalho, Núria Casacuberta-Barberà, Manuel Castellà, Andrea Castellví, Pedro Castro, Ramon Cicuendez Ávila, Catia Cillóniz, Luisa Clar, Cristina Climent, Jordi Codina, Pamela Conde, Sofía Contreras, María Cruz Martin, Raul de Pablo Sánchez, Diego De Mendoza,

Cecilia del Busto Martínez, Yolanda Díaz, María Digna Rivas Vilas, Cristina Dólera Moreno, Irene Dot, Pedro Enríquez Giraudo, Inés Esmorís Arijón, Teresa Farre Monjo, Javier Fernández, Carlos Ferrando, Albert Figueras, Eva Forcadell-Ferreres, Lorena Forcelledo Espina, Nieves Franco, Àngels Furro, Felipe García, Beatriz García, Emilio García Prieto, Carlos García Redruello, Amaia García Sagastume, Maria Luisa Gascón Castillo, Gemma Gomà, Vanesa Gómez Casal, Silvia Gómez, Carmen Gómez Gonzalez, Jessica González, Federico Gordo, Maria Pilar Gracia, Alba Herraiz, Rubén Herrán-Monge, Mercedes Ibarz, Silvia Iglesias, Maria Teresa Janer, Gabriel Jiménez, Mar Juan Díaz, Karsa Kiarostami, Juan I Lazo Álvarez, Miguel León, Alexandre López-Gavín, Ana López Lago, Desire Macias Guerrero, Nuria Mamolar Herrera, Rafael Mañez Mendiluce, Cecilia L Mantellini, Gregorio Marco Naya, Pilar Marcos, Enrique Marmol Peis, Paula Martín Vicente, María Martínez, Carmen Eulalia Martínez Fernández, Maria Dolores Martínez Juan, Juan Fernando Masa Jimenez, Joan Ramon Masclans, Emilio Maseda, Eva María Menor Fernández, Mar Miralbés, Josman Monclou, Juan Carlos Montejo-González, Neus Montserrat, María Mora Aznar, Pedro Moral-Parras, Dulce Morales, Sara Guadalupe Moreno Cano, David Mosquera Rodríguez, Rosana Muñoz-Bermúdez, José María Nicolás, Ramon Nogue Bou, Rafaela Nogueras Salinas, Marta Ocón, Ana Ortega, Sergio Ossa, Pablo Pagliarani, Francisco Parrilla, Leire Pérez Bastida, Purificación Pérez, Gloria Pérez Planelles, Eva Pérez Rubio, David Pestaña Laguna, Àngels Piñol-Tena, Javier Prados, Andrés Pujol, Núria Ramon Coll, Gloria Renedo Sanchez-Giron, Ferran Roche-Campo, Laura Rodriguez, Felipe Rodríguez de Castro, Silvia Rodríguez, Covadonga Rodríguez Ruiz, Jorge Rubio, Alberto Rubio López, Miriam Ruiz Miralles, Pablo Ryan Murúa, Eva Saborido Paz, Ana Salazar Degracia, Inmaculada Salvador-Adell, Miguel Sanchez, Ana Sánchez, Bitor Santacoloma, Maria Teresa Sariñena, Marta Segura Pensado, Lidia Serra, Mireia Serra-Fortuny, Ainhoa Serrano Lázaro, Lluís Servià, Laura Soliva, Carla

Speziale, Adrián Tormos, Gerard Torres, Mateu Torres, Sandra Trefler, Javier Trujillano, Alejandro Úbeda, Luis Urrelo-Cerrón, Estela Val, Luis Valdivia Ruiz, Montse Vallverdú, Maria Van der Hofstadt Martin-Montalvo, Sabela Vara Adrio, Nil Vázquez, Javier Vengoechea, Clara Vilà-Vilardel, Judit Vilanova, Tatiana Villada Warrington, Hua Yang, Minlan Yang, Ana Zapatero
